# Disgust, Contagion Beliefs, and Contamination-Related Obsessive-Compulsive Symptoms in Hospital and Non-Hospital Workers

**DOI:** 10.3390/jcm15218452

**Published:** 2026-10-30

**Authors:** Lars Oliver Martin Rothkegel, Nicolás Reinoso Schiller, Thamea Winterfeld, Stefan Bushuven, Simone Scheithauer, Jakob Fink-Lamotte

**Affiliations:** 1 Department of Psychology, Clinical Psychology, University of Potsdam, Karl-Liebknechtstraße 24/25, 14476 Potsdam, Germanyjakob.fink-lamotte@uni-potsdam.de (J.F.-L.); 2 Department for Infection Control and Infectious Diseases, University Medical Center Göttingen, Georg-August University Göttingen, 37075 Göttingen, Germany; 3 Institute for Infection Control and Infection Prevention, Health Care Association District of Constance, 78224 Singen, Germany

**Keywords:** obsessive-compulsive disorder, contamination, disgust, hospital workers, occupational exposure, contagion beliefs, hospital personnel

## Abstract

**Background:** Recent research highlights the role of disgust in obsessive-compulsive disorder (OCD), particularly contamination-related OCD (C-OCD). Beyond elevated trait disgust, individuals with C-OCD often endorse strong beliefs about the spread of contamination through contact (contagion beliefs). Hospital workers (HWs) operate in environments where contamination is part of daily life and must manage genuine infection risks and their own disgust responses. **Methods:** To examine whether hospital work and medical training may confer resilience relevant to C-OCD, disgust and contagion beliefs (confirmatory outcomes), as well as OCD symptoms and situation-specific contamination concerns (exploratory outcomes), were assessed using self-report questionnaires in an online cross-sectional study. Participants included psychology students (N = 52), medical students (N = 69), non-medical HWs (N = 44), and medical HWs (N = 111). **Results:** We hypothesized that medical HWs would report the lowest levels of disgust and contagion beliefs, followed by medical students, non-medical HWs, and psychology students. This hypothesis was partially supported for trait disgust only. Contrary to expectations, HWs, regardless of medical background, reported the highest levels of contagion beliefs. Despite these elevated beliefs, HWs reported significantly lower contamination concerns than psychology students. Moreover, contagion beliefs were not associated with C-OCD symptoms among medical HWs, whereas this association was present in all other groups. **Conclusions:** Contagion beliefs may not be associated with C-OCD symptoms in the same way across contexts, particularly among individuals who regularly manage genuine contamination threats. Future research should investigate which factors may buffer HWs against the typically maladaptive association between contagion beliefs and C-OCD symptoms.

## 1. Introduction

Contamination-related obsessive-compulsive disorder (C-OCD) is frequently identified as the most prevalent obsessive-compulsive disorder (OCD) subtype (e.g., ref. [1]). Although well-established and empirically supported treatments are available (with exposure and response prevention (ERP) therapy considered the gold standard for treating OCD [2]), about 30% of individuals show little to no improvement [3]. Over the past two decades, research has identified disgust as a key factor in the development and maintenance of OCD [4,5], suggesting that it may play a crucial role in successful treatment (e.g., refs. [6,7,8]). ERP operates, among other mechanisms, through expectancy violation, inhibitory learning, and habituation [9]. However, this is precisely where the difficulty in treating C-OCD appears to lie, because previous studies have shown that exposure-based interventions reduce disgust responses less strongly and more slowly than fear responses [10,11]. Several mechanisms may account for this specificity. The following four illustrate key possibilities (for a German review, see [7]).

(1) Disgust associations often develop through evaluative conditioning, whereby stimuli acquire a stable negative valence that is less susceptible to change through expectancy violation or habituation (e.g., refs. [12,13]).
(2) Expectancy violations are more difficult to experience in the context of disgust, as negative consequences (e.g., illness) tend to occur with a temporal delay and are not immediately verifiable (e.g., ref. [8]).
(3) Disgust is frequently accompanied by attentional avoidance, which may limit key learning processes during exposure (e.g., ref. [14]).
(4) Finally, specific cognitive biases such as the “laws of sympathetic magic,” particularly the “law of contagion,” play an important role. Individuals with C-OCD show pronounced beliefs about transfer, which contribute to an overestimation of a contamination risk [15,16] and thus reinforce avoidance and cleansing behaviors [17].

While all the above discussed mechanisms presumably play an important role, this study will focus on aspects 2 and 4, the potential temporal delay of safety signals in disgust exposure as well as the impact of contagion beliefs on C-OCD symptomatology and its relation to disgust.

Contagion beliefs implicate that contamination persists and spreads through contact, even when no objective threat is present. This assumption is consistent with the law of contagion, which proposes that once two objects come into contact, properties of one object are believed to transfer to the other and to persist even after contact has ended (e.g., a clean shirt touching a contaminated surface is perceived as permanently contaminated; ref. [15]). In C-OCD, these beliefs maintain fear and avoidance and consequently may reduce the effectiveness of ERP, because patients continue to perceive contamination as persistent despite repeated exposure to objectively safe situations.

Taken together, these two mechanisms suggest a joint explanation for why ERP may be less effective in C-OCD: expectancy violations regarding disgust-based threats are difficult to experience because negative consequences (e.g., illness) often occur with a delay and cannot be immediately verified as absent, while contagion beliefs assume that contamination persists through contact even without objective threat—thereby maintaining fear and avoidance despite repeated safe exposure. Hospital settings offer a naturalistic model for examining whether repeated, long-term exposure to contamination-relevant cues—without the time limit typical of ERP protocols—can provide the kind of repeated, verifiable safety signal that classical exposure interventions struggle to deliver. Hospital workers are routinely confronted with cues related to illness, pathogens, and infection, giving them repeated opportunities to learn, over time, that contact with such cues does not often result in the feared consequences [18].

Consistent with this model, healthcare workers and students consistently show lower disgust propensity (DP) than other populations [19,20,21,22], an effect that increases with professional experience [20,23,24,25] and age [19]. This pattern has been attributed to habituation [20], self-selection effects [22,26,27], or the development of coping strategies that offset frequent disgust-eliciting experiences [18]. At the same time, high disgust remains associated with emotional exhaustion and stronger intentions to quit among hospital workers [28], and more than half of nursing students report disgust experiences at work severe enough to make them consider leaving the profession [29]. While this evidence indicates that DP decreases with sustained occupational exposure, it remains unknown whether the same pattern extends to contagion beliefs, situation-specific contamination concerns, and C-OCD symptoms, or how these constructs relate to one another across groups. This gap is also theoretically relevant: it is unclear whether elevated contagion beliefs in C-OCD reflect primary appraisal (how contagious a stimulus is judged to be) or secondary appraisal (how well one believes one can cope with that contagiousness; ref. [30]). Distinguishing contagion beliefs (primary appraisal) from contamination concerns (a combination of primary and secondary appraisal; ref. [31]) helps address this: lower contagion beliefs in hospital workers would indicate reduced primary appraisal, whereas unchanged contagion beliefs alongside reduced contamination concerns would instead point to more effective coping with perceived infection risk. The present study addresses this gap by comparing hospital workers with a medical background (i.e., involved in direct patient care) to three comparison groups—hospital workers without a medical background, medical students, and psychology students—on DP, contagion beliefs, contamination concerns, and C-OCD symptoms. If long-term hospital workers show lower contagion beliefs and contamination concerns than medical or psychology students, this would suggest that the repeated, real-world experience of not becoming infected constitutes a stronger safety signal than the time-limited exposure typical of ERP.

On this basis, our preregistered hypotheses (see Section 2.1) were that hospital staff with a medical background would show (1) lower levels of DP and (2) lower levels of contagion beliefs than non-medical hospital staff, medical students, and psychology students. Medical hospital workers and psychology students were chosen as two groups differing strongly in their current and past exposure to potential pathogens as well as in patient contact and disease-related knowledge. Medical students and non-medical hospital workers were included to disentangle two aspects of this exposure: Medical students share medical knowledge and an interest in diseases, and typically have some patient contact through clinical training, but have less sustained pathogen exposure than medical staff. Non-medical hospital workers (e.g., administrative or technical staff) encounter potential pathogens in their everyday work environment, but have no direct patient contact and no medical training. Comparing these groups therefore allows us to test whether lower contagion beliefs and DP are related to occupational pathogen exposure, medical knowledge, or both. Based on the frequency of current and past patient contact and pathogen exposure, we expected the following order: medical hospital workers (most exposure), followed by non-medical hospital workers and medical students, and finally psychology students (least exposure).

As additional, non-preregistered variables, C-OCD symptomatology and contamination concerns were assessed to further characterize the relationship between psychopathology, contagion beliefs, disgust, and situational contamination concerns. Given that contagion beliefs, contamination concerns, and DP have all been linked to C-OCD symptomatology, the relationship between these constructs and C-OCD symptoms was additionally examined separately as an exploratory analysis.

## 2. Materialsand Methods

### 2.1. Participants

The study, including the power analysis, was preregistered (AsPredicted #232,767; https://aspredicted.org/f9b4-v37c.pdf (preregistred on 10 June 2025)). In summary, the planned sample consisted of 32 participants in each comparison group (i.e., psychology students, medical students, and hospital workers without a medical background) and 96 hospital workers with a medical background. This sample size corresponds to a statistical power of 0.80 for detecting significant results with an effect size of d=0.5, which is approximately the effect size reported by Edgar et al. [20].

Participation in the study was voluntary. Recruitment was conducted between 15 July and 15 October 2025; two reminders were sent in August and September to encourage participation. Convenience sampling was applied in accordance with the two-centre recruitment design: Medical students and hospital staff with a medical background were recruited at one centre (University Medicine Göttingen), while psychology students were recruited at another centre (University of Potsdam). Psychology students were able to obtain course credit for participation. The survey link was distributed via staff and student multipliers, internal communication channels, and student social media groups (e.g., WhatsApp). Owing to the open and untracked dissemination of the link and limited response activity, the number of individuals reached could not be determined; consequently, an exact response rate could not be calculated.

Participants were eligible if they (a) provided informed consent, (b) were medical or psychology students (currently enrolled, with no restriction regarding academic year) or hospital workers (HWs) with or without medical background, (c) were at least 18 years old, (d) reported no neurological disorder or addiction to psychoactive substances (except caffeine or nicotine), and (e) completed the study in a quiet environment or using headphones. Participants were excluded if they answered fewer than 70 % of the questions, reported an attention level of 50 % or lower, had a within-person standard deviation <0.5 across all DP items, had a longstring index more than 3 SD above the mean on the DP questionnaire, did not provide an answer about group assignment or did not complete the DP questionnaire. In total, 561 participants started the study by clicking on the study link, of whom 339 completed it. Of these, 319 answered at least 70% of the questions, and 289 reported an attention level above 50% while completing the questionnaires. Three participants were excluded because of a high longstring index on the DP questionnaire, and one because of a very small within-person standard deviation (<0.5) across the DP items. One further participant was excluded because the question on group assignment was not answered. Of the remaining 284 participants, 5 had not completed the DP questionnaire, and 3 could not be reliably assigned to a group: they selected “other” and specified their occupation as “biologist”, “infection biologist”, and “student”, and also reported not working in a hospital. The final sample comprised 276 participants: 111 HWs with medical background, 44 HWs without medical background, 69 medical students, and 52 psychology students. Table 1 shows the distribution of gender and age for all four groups. All group sizes exceeded the minimum sample size (N = 32 for control groups and N = 96 for the comparison group) determined by the power analysis.

### 2.2. Materials

DP was assessed using the German version of the Disgust Scale (FEE; Fragebogen zur Erfassung der Ekelempfindlichkeit; refs. [32,33]). This validated questionnaire consists of 37 items spanning 5 distinct disgust domains: death, body secretions, hygiene, spoilage, and oral rejection. The scale exhibits excellent internal consistency overall (original, α=0.9; this study, α=0.94), with domain-specific reliabilities ranging from α=0.69 for oral rejection (this study, α=0.77) to α=0.85 for death (this study, α=0.68).

Contamination concerns were assessed using the Multidimensional State Disgust Questionnaire (MSDQ, ref. [31]). The MSDQ is a situation-specific assessment tool, designed to measure the four dimensions of physical, moral, self disgust, and contamination concerns. For this study, only the contamination concern scale was investigated. The 4-factor structure of the 18-item MSDQ was confirmed via exploratory and confirmatory factorial analysis; the internal consistency of the 4-item-contamination concern scale was high, with α=0.84.

Contagion beliefs were assessed using the video-based Chain of Contagion Task (CCT; ref. [16], originally developed by Tolin et al. [15]). The CCT measures perceived contamination across multiple degrees of separation. In the task, a neutral pencil is initially brought into contact with a disgust-eliciting stimulus and is then used to sequentially contaminate additional pencils, such that the first pencil contacts the second, the second contacts the third, and so forth, until the twelfth pencil in the original paradigm. For each pencil, participants rated perceived contamination. All ratings were provided on a scale ranging from 0 (not at all) to 100 (very much). In contrast to the original version of the task, only nine pencils were used in the present study. This modification was based on prior findings from correlational analyses indicating negligible interindividual variability beyond the eighth pencil and no additional informational value beyond the tenth pencil [34].

Obsessive-compulsive symptom severity was assessed using the Dimensional Obsessive-Compulsive Scale (DOCS; ref. [35]), a 20-item self-report measure that assesses overall OCD severity across four symptom dimensions: contamination, responsibility, unacceptable thoughts, and symmetry. Each dimension is evaluated across five severity parameters (time spent, avoidance, distress, functional interference, and resistance) using a 5-point scale ranging from 0 (not at all) to 4 (extremely). The German version of the DOCS [36] demonstrates excellent internal consistency (Cronbach’s α≥0.90) and good test–retest reliability (r≈0.80), as well as satisfactory construct validity and diagnostic accuracy; criterion validity is reported to be limited. In the present study, only the five items from the contamination dimension (C-DOCS) were assessed.

### 2.3. Study Procedure

**Figure 1:**
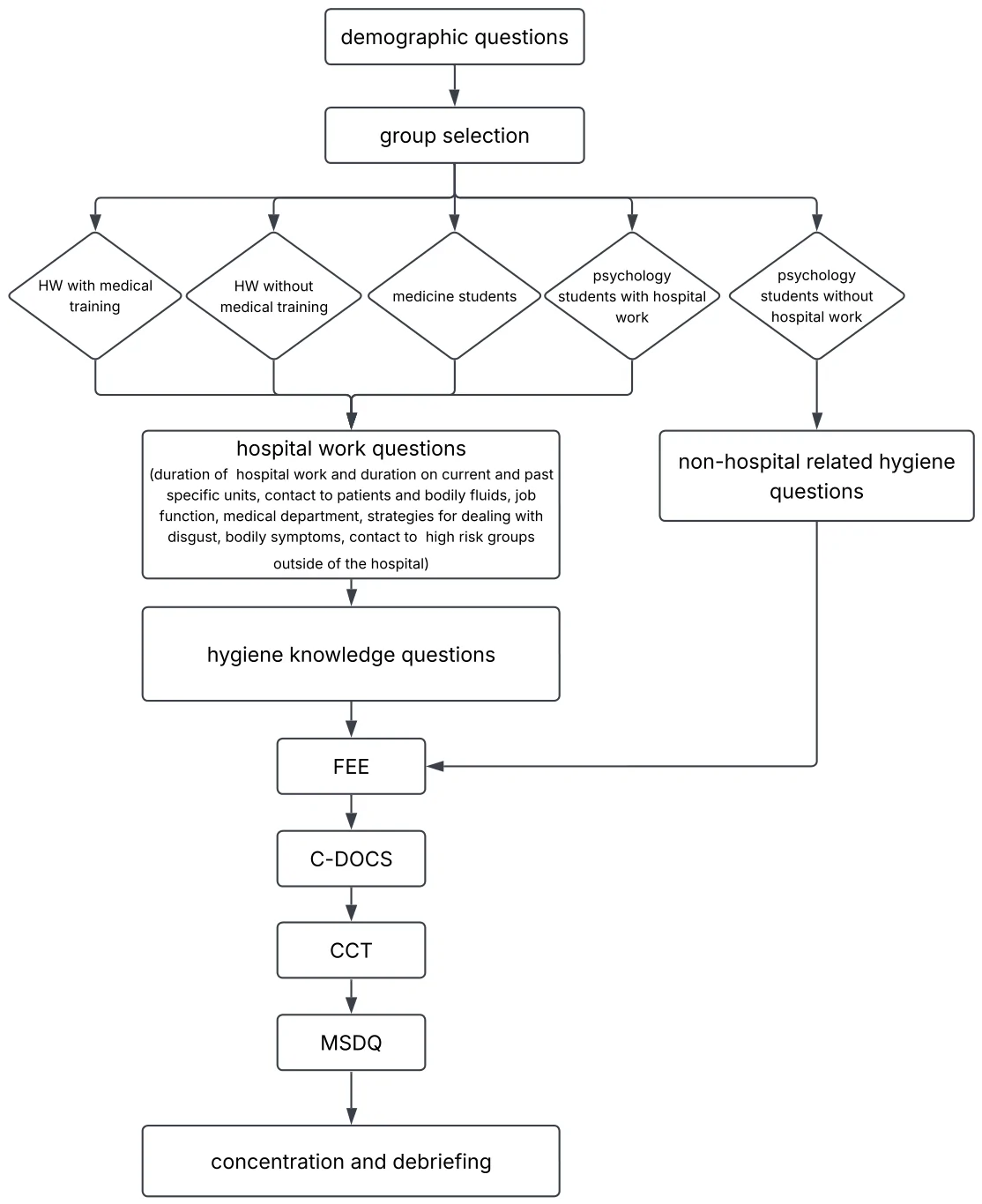
Flowchart depicting the study procedure. HWs = Hospital Workers; FEE = Disgust Propensity Questionnaire (Fragebogen zur Erfassung der Ekelempfindlichkeit); C-DOCS = Contamination Subscale of the Dimensional Obsessive-Compulsive Scale; CCT = Chain of Contagion Task; MSDQ = Multidimensional State Disgust Questionnaire.

The study was conducted entirely online and in German. Participants were instructed to complete an online version of the CCT along with a series of questionnaires assessing relevant hygienic and psychological aspects: DP, contamination concerns, contagion beliefs, and C-OCD-symptom severity, as well as approximately ten additional items regarding participants’ professional experience in the hospital setting (e.g., “How long have you been working in the hospital?” and “How would you rate your own knowledge regarding hospital hygiene?”). Additionally, all participants were instructed to think of a disgusting situation within a hospital (or, if they did not remember one, outside a hospital) and afterwards answered the MSDQ. The complete flowchart, including all questionnaires and questions, is presented in Figure 1. Mean of the total participation time was 25.6 min (SD=8.94). For group assignment, each participant indicated whether they were a psychology student, a medical student, a non-medical hospital worker, a medical hospital worker, or other. Participants who selected “other” and specified “student of dentistry” were assigned to the medical student group. All “other” responses were inspected individually; these participants were either assigned to the corresponding group or excluded from the sample (see above Section 2.1).

### 2.4. Data Preparation and Statistical Analysis

For the dependent variables, mean scores of all 37 items of the FEE were calculated to assess DP; the aggregated score of the question about contamination level for each of the 9 successive pens within the CCT was used to assess contagion beliefs; the mean of the 4 items of the respective subscale of the MSDQ was calculated to estimate contamination concerns; and the mean score of the 5 items of the C-DOCS were computed to assess C-OCD symptom severity.

Visual inspection of Q-Q plots indicated moderate deviations from normality in the tails of the residual distributions for the contagion beliefs, contamination concerns, and C-DOCS models, likely reflecting floor-and-ceiling effects inherent to these bounded scales, along with a small number of correspondingly extreme observations; residuals of the FEE model showed close alignment with the theoretical normal distribution across most of the range. Given the sample size (N = 276, with group sizes exceeding 40), these deviations are unlikely to compromise the validity of the parameter estimates, as the central limit theorem supports approximately normal sampling distributions of the coefficient estimates regardless of residual non-normality. To guard against potential heteroscedasticity, all models were additionally estimated using heteroscedasticity-consistent standard errors (HC3; ref. [37]). Across all models, results were substantively unchanged compared to conventional OLS standard errors.

To compare the expression of the dependent variables across groups, separate linear models were computed for each dependent variable, with group as a predictor variable. In all models, HWs with medical background served as the reference category against which all other groups were compared, because this group was the main focus within the hypotheses. Also, due to previous findings indicating lower disgust for men [33] and, to a lesser extent, older participants [38], the models were always controlled for age and gender. The variable age was mean-centred prior to analysis by subtracting the sample mean (M=34.9), while the original unit (years) was retained. Mean-centring was applied to facilitate the interpretation of the intercept, which consequently represents the expected outcome at the mean age of the sample. Gender was included as a categorical predictor with three levels (male, female, non-binary), with male as the reference category. Two participants identified as non-binary; given this small subgroup size, the corresponding coefficient is not meaningfully interpretable in any model and is therefore not discussed in the main text. Thus, each linear model contained group (with the four levels of occupation), age and gender as predictor variables. Firstly, the models were computed in full and then post hoc pairwise comparisons were computed for a more detailed understanding of group comparisons.

Regarding the relationship between the dependent variables and C-OCD symptomatology, a linear model was computed with C-OCD symptoms as the dependent variable, along with contagion beliefs, group, and their interaction as predictor variables, controlling for contamination concerns and DP as covariates. Age and gender were included as additional covariates in the model. Given the conceptual overlap between contagion beliefs, contamination concerns, and DP, variance inflation factors (VIFs) were inspected to assess multicollinearity among predictors; all values for non-interaction terms were below 2 (${\mathrm{GVIF}}^{1/(2\cdot df)}$), indicating no problematic multicollinearity. All statistical analyses were conducted in R (version 4.4.3; ref. [39]) using the dplyr, effectsize, car, emmeans, sandwich, and lmtest packages [40,41,42,43,44].

## 3. Results

### 3.1. Group Differences in Dependent Variables

**Figure 2:**
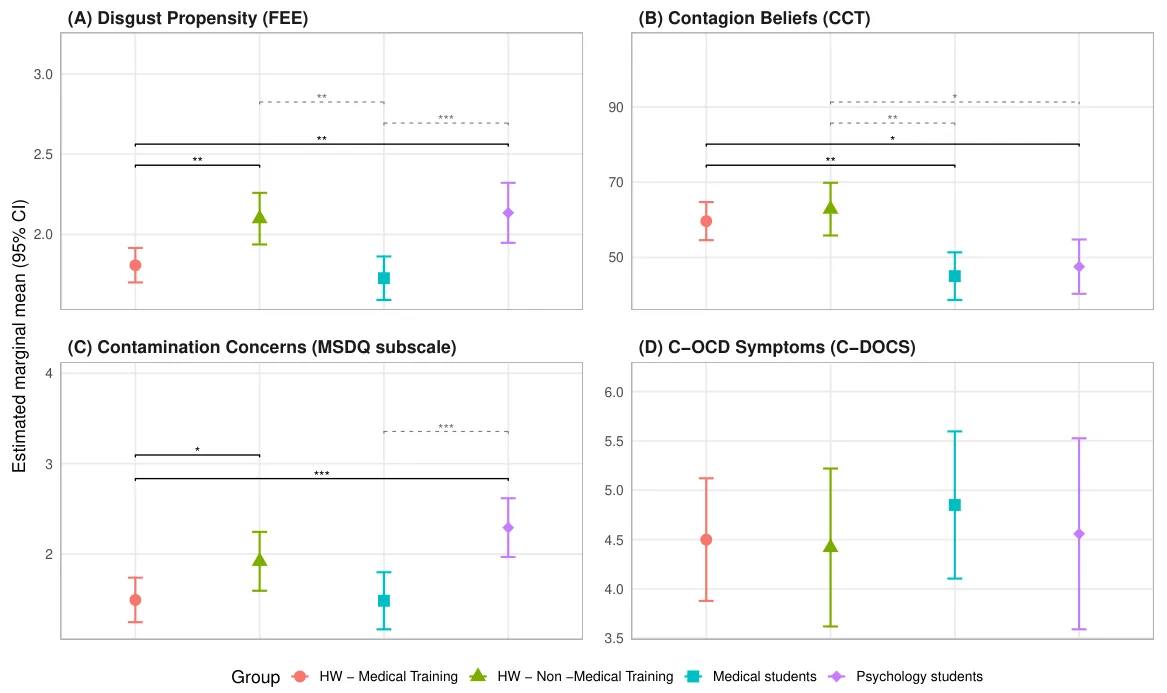
Estimated marginal means (adjusted for age and gender; averaged over gender proportionally to sample composition) for each group on (**A**) disgust propensity (DP), measured with the Fragebogen zur Erfassung der Ekelempfindlichkeit (FEE); (**B**) contagion beliefs, measured with the Chain of Contagion Task (CCT); (**C**) contamination concerns, measured with the corresponding subscale of the Multidimensional State Disgust Questionnaire (MSDQ); and (**D**) contamination-related obsessive-compulsive disorder (C-OCD) symptoms, measured with the Contamination Subscale of the Dimensional Obsessive-Compulsive Scale (C-DOCS). Error bars represent 95% confidence intervals based on heteroscedasticity-consistent (HC3) standard errors. Solid brackets: Comparisons involving HWs with medical training (unadjusted; preregistered for panels (**A**,**B**), exploratory for panels (**C**,**D**)). Dashed grey brackets: Exploratory pairwise comparisons between the remaining groups (Tukey-adjusted). Only significant comparisons are shown: * p<0.05, ** p<0.01, *** p<0.001. Y-axes are scaled separately for each panel.

The results of the group comparisons for all dependent variables are presented in Figure 2. The following paragraphs summarize these findings separately for each dependent variable. Effect sizes, standard error of the mean, as well as F-, t-, and *p*-values are reported for statistically significant comparisons. It should be noted that analyses concerning contagion beliefs and DP were preregistered, whereas analyses involving C-OCD symptoms and contamination concerns were not.

#### 3.1.1. Disgust Propensity

Mean DP measured with the FEE for all four groups can be seen in Figure 2a. A linear regression with HC3-robust standard errors was conducted predicting DP from group membership, age, and gender ($R^{2}=0.17$). As hypothesized, HWs with medical training reported significantly lower DP than psychology students (β=−0.33, SE=0.12, t=−2.75, p=0.006, d=−0.63) and HWs without medical training (β=−0.29, SE=0.09, t=−3.11, p=0.002, d=−0.56). However, no significant difference between medical students and HWs with medical training could be observed (p=0.41). Women reported significantly higher DP than men (β=0.35, SE=0.07, t=4.83, p<0.001), while age was not a significant predictor (β=−0.005, SE=0.003, t=−1.56, p=0.119). Exploratory Tukey-corrected pairwise comparisons further revealed that medical students also reported significantly lower DP than psychology students (Δ=0.41, t=3.89, p<0.001, d=−0.78) and HWs without medical training (Δ=0.37, t=3.28, p=0.006, d=0.71). HWs without medical training and psychology students did not differ significantly (p=0.99).

#### 3.1.2. Contagion Beliefs

A linear regression with HC3-robust standard errors was conducted predicting contagion beliefs from group membership, age, and gender ($R^{2}=0.05$). Contrary to the hypotheses, HWs with medical training reported significantly higher contagion beliefs than psychology students (β=12.12, SE=4.97, t=2.44, p=0.015, d=0.53). Additionally, HWs with medical training also reported significantly higher contagion beliefs than medical students (β=14.64, SE=4.58, t=3.20, p=0.002, d=0.64), while HWs without medical training did not differ significantly from HWs with medical training (p=0.44). Age was a significant negative predictor (β=−0.37, SE=0.15, t=−2.56, p=0.011), while gender was not significant (p=0.68). Exploratory Tukey-corrected pairwise comparisons revealed that medical students (Δ=17.84, t=3.49, p=0.003, d=−0.78) as well as psychology students (Δ=15.31, t=2.81, p=0.027, d=−0.67) reported significantly lower contagion beliefs than HWs without medical training. Medical students and psychology students did not differ from each other (p=0.936).

#### 3.1.3. Contamination Concerns

A linear regression with HC3-robust standard errors was conducted predicting contamination concerns from group membership, age, and gender ($R^{2}=0.15$). HWs with medical training reported significantly lower contamination concerns than psychology students (β=−0.80, SE=0.23, t=−3.43, p<0.001, d=−0.75) and HWs without medical training (β=−0.43, SE=0.18, t=−2.31, p=0.021, d=−0.40). Age was a significant negative predictor (β=−0.016, SE=0.006, t=−2.53, p=0.012), while gender was not significant (p=0.203). Exploratory Tukey-corrected pairwise comparisons further revealed that medical students also reported significantly lower contamination concerns than psychology students (Δ=−0.81, t=−3.91, p<0.001, d=−0.75). No further significant differences emerged between any other group pairs (all p≥0.098). The differences in contamination concerns is displayed in Figure 2c.

#### 3.1.4. C-OCD Symptoms

A linear regression with HC3-robust standard errors was conducted predicting C-OCD-related contamination symptoms (C-DOCS) from group membership, age, and gender ($R^{2}=0.04$). Group membership did not significantly predict C-DOCS scores, with none of the groups differing from HWs with medical training (all p≥0.53). Age was a significant negative predictor (β=−0.032, SE=0.015, t=−2.06, p=0.040), while gender did not predict the C-DOCS score significantly (p=0.305). Exploratory Tukey-corrected pairwise comparisons confirmed the absence of group differences, with all pairwise contrasts being non-significant (all p≥0.887).

### 3.2. The Influence of Contagion Beliefs on C-OCD Symptoms

**Figure 3:**
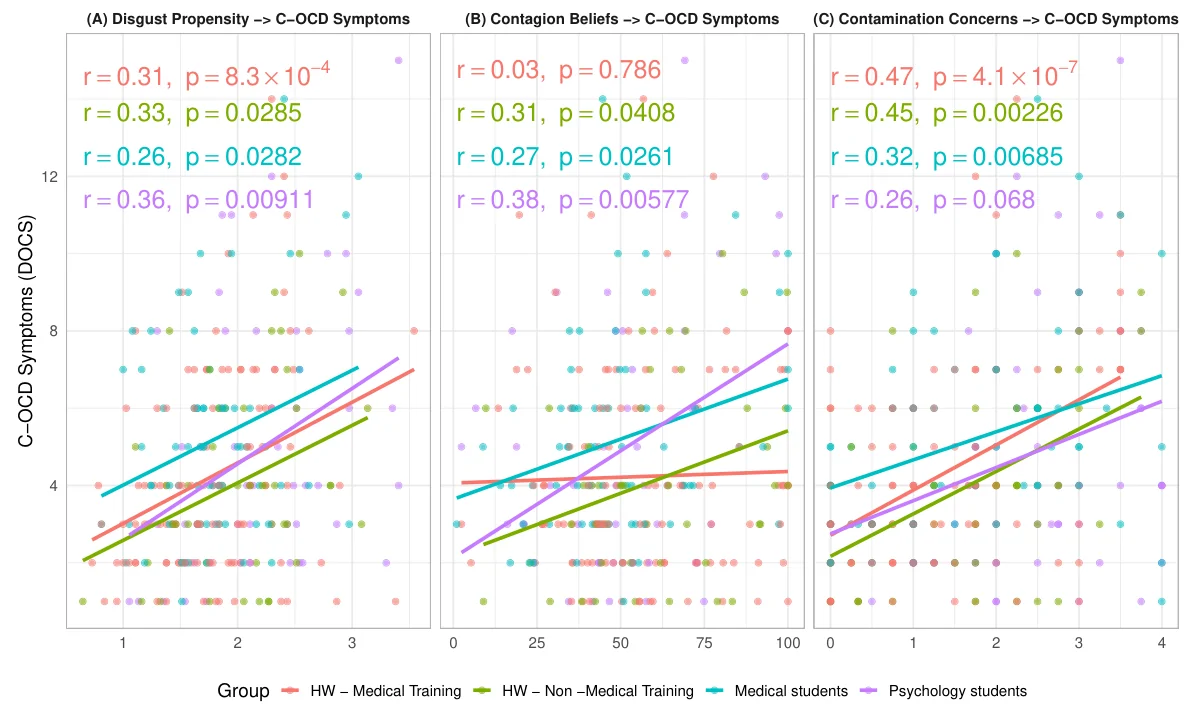
The relationship between (**A**) disgust propensity, (**B**) contagion beliefs, and (**C**) contamination concerns with C-OCD symptoms for all four observation groups.

Given that the findings regarding contagion beliefs were contrary to the hypothesized direction, whereas the pattern for DP was consistent with the hypotheses and the exploratory findings for contamination concerns followed a similar direction as DP, differences in the influence of contagion beliefs on C-OCD symptoms between hospital workers and the other groups were examined. As indicated in the introduction, increased contagion beliefs alongside decreased contamination concerns suggest that, for hospital workers, heightened contagion beliefs are not necessarily problematic and should therefore not contribute to C-OCD symptomatology. Thus, while previous studies have shown an association between contagion beliefs and C-OCD symptoms [15,16], the analyses indicated that this relation might not be present in hospital workers. This rationale is based on the assumption that extensive knowledge of, and diverse experiences with, contaminating stimuli enhance hospital workers’ self-efficacy without altering their beliefs themselves. Figure 3 shows the influence of DP, contagion beliefs, and contamination concerns on C-OCD symptoms across the four groups in separate colors. None of these analyses were preregistered and thus are all exploratory.

A model containing age, gender, group, contamination concerns, and FEE as covariates, along with the interaction between group and contagion beliefs, significantly improved fit over the corresponding main-effects model (Wald test: F(3,253)=3.05, p=0.029). Specifically, the interaction between contagion beliefs and group was significant when comparing HWs with medical background to psychology students (β=0.06, SE=0.02, t=2.60, p=0.010), indicating that the positive association between contagion beliefs and C-OCD symptoms was significantly weaker in HWs with a medical background compared to psychology students. A similar, non-significant trend was observed when comparing HWs with medical background to medical students (β=0.03, SE=0.02, t=1.65, p=0.101) and to HWs without medical training (β=0.03, SE=0.02, t=1.54, p=0.124). Grouping participants by medical background alone (medical vs. non-medical) confirmed this pattern, with a significant contagion beliefs × medical background interaction (Wald test: F(1,257)=6.01, p=0.015).

### 3.3. Summary of Results

In sum, disgust propensity and contamination concerns were lower in the medically trained groups, whereas contagion beliefs were highest in HWs, and C-OCD symptoms did not differ between groups. The association between contagion beliefs and C-OCD symptoms was weaker in HWs with a medical background than in psychology students.

## 4. Discussion

In an online study, we examined disgust propensity (DP), contagion beliefs, contamination concerns, and C-OCD symptoms in hospital workers (HWs) with and without a medical background, medical students, and psychology students. The key finding was a dissociation in medically trained HWs: contagion beliefs were elevated, whereas contamination concerns were low and the association between contagion beliefs and C-OCD symptoms was weaker than in psychology students.

### 4.1. Summary and Interpretation of Confirmatory Analysis

Partially confirming Hypothesis 1, DP was lower for HWs with medical background than for psychology students and HWs without medical background. However, medical students did not differ from HWs with medical background. This is in line with previous studies [26,27], supporting the notion that DP acts as a robust predictor of career selection. The absence of a difference in DP between medical students and HWs with a medical background is also relevant to the treatment of C-OCD. Notably, HWs with a medical background had worked in hospital settings between 2 and 48 years (M = 19.6 years), suggesting that even prolonged occupational exposure to potentially disgust-evoking stimuli may not be associated with substantially lower DP. This interpretation is consistent with previous evidence indicating that disgust is relatively resistant to habituation through mere exposure [4,45]. One study of healthcare assistants in care homes [20] found a reduction in disgust domains specific to the healthcare environment, with some evidence for generalization beyond it. This contrasts with the absence of a difference between HWs and medical students in the present study, and points to a need for further research on how DP changes over time in hospital work. Age has previously been negatively correlated with DP (e.g., ref. [19]), yet age was not a significant predictor of DP here, despite a larger sample than that of Ousey et al. [19]. Sample characteristics may also explain the diverging findings: Edgar et al. [20] studied healthcare assistants, whereas the young comparison group here consisted exclusively of medical students and did not include, for example, nursing students. One possible explanation is that habituation to disgust, and the associated decline in trait disgust, occurs early in medical education and then remains relatively stable. Rozin [24], for instance, found a rapid decline in disgust toward death and body-envelope violations within only a few months of medical school. Similarly, Adagide et al. [23] found a decline in disgust across the years of nursing school. However, these studies were either short-term or cross-sectional. Longitudinal studies tracking medical students’ DP before, during, and after training would help clarify this trajectory.

A surprising result, contradictory to H2, was that HWs with a medical background showed higher contagion beliefs than psychology students, despite their lower DP. This effect was specific to HWs, since medical students did not show elevated contagion beliefs. One explanation is that contact precautions, used daily in hospitals to prevent cross-transmission of pathogens such as multi-drug-resistant bacteria, keep the contact transmission route salient for HWs. Because the result regarding contagion beliefs was surprising, additional exploratory analyses were conducted.

### 4.2. Summary and Interpretation of Exploratory Analysis

The exploratory results revealed that within HWs with a medical background, contagion beliefs were unrelated to C-OCD symptoms and contamination concerns were very low. This is notable because contagion beliefs have been identified as an important factor in the development and maintenance of C-OCD, and are targeted by several disgust-focused OCD treatments (e.g., ref. [46]). The present results suggest that professional experience in an environment where contamination is either constantly present or a frequent, actively managed risk may break the link between contagion beliefs and C-OCD symptoms. This points to a hypothesis for future intervention studies: contagion beliefs may not be inherently maladaptive; instead, it may be the appraisal of contamination and one’s perceived ability to cope with it that determines whether these beliefs become clinically relevant.

### 4.3. Implications for the Treatment of Disgust

Returning to the four processes proposed to reduce the effectiveness of exposure therapy in C-OCD (see [7]), the present results speak to two of them. First, strong contagion beliefs, as measured via performance on the CCT [15], were not necessarily associated with C-OCD symptoms among individuals with extensive experience and knowledge in managing pathogens. Second, regarding the absence of expectancy violation, HWs with many years of workplace experience did not differ in DP from medical students. Therefore, within this sample, long-term workplace exposure was not associated with lower disgust. Exposure therapy might therefore benefit more from directly targeting expectancies, for example, by focusing on promoting and increasing self-efficacy (e.g., “I will [not] be able to stand this amount of disgust”; see [47]), than from simply extending exposure time—though further studies are needed to confirm this.

### 4.4. Limitations and Strengths of This Study

One strength of this study is its comparatively large number of hospital workers, including both those with and without a medical background, exceeding the sample size estimated in the power analysis. Recruiting hospital staff is often difficult given their demanding schedules and limited time for research participation. However, participation was voluntary: of the 561 participants who started the study, only 319 finished it, and those with a particular interest in disgust, contamination, or related topics may have been more likely to complete it, introducing a possible self-selection bias. A further strength is the study’s interdisciplinary approach, which combines psychological and medical perspectives on contamination-related processes. This integration may help clarify how contamination-related beliefs and responses matter both for psychological symptoms and for infection prevention and control in healthcare settings. The findings on contamination concerns, and their relationship to profession and C-OCD symptoms, were exploratory. Because the pattern of results for contagion beliefs was completely reversed relative to the hypothesized direction, a new hypothesis was formulated before running the corresponding analyses, but without preregistration; replicating these results is therefore necessary to strengthen their impact. For contamination concerns, participants generated their own contamination-relevant situation. This likely produced more individually valid, idiosyncratic disgust responses, but at the cost of standardization and, potentially, reliability; replicating the study with standardized hospital situations could strengthen these results further. Groups also differed considerably in age, reflecting the natural age distribution within each group. The sample was over 70% female, which is broadly representative of the gender distribution within the professions studied; all analyses were controlled for age and gender to reduce bias from these demographic differences. The final analysis deviated slightly from the preregistered group classification. The preregistration specified that hospital staff with a medical background and patient contact would show lower disgust and contagion beliefs than the other groups. However, patient contact could not be operationalized unambiguously: current patient contact was assessed for participants currently working in a hospital, while previous patient contact was assessed for those who had worked in a hospital in the past. As a result, several participants reported currently working in a hospital without patient contact despite substantial previous patient contact. The final analysis therefore used four groups instead—hospital workers with a medical background, hospital workers without a medical background, medical students, and psychology students—deviating from the preregistration. As indicated above, the cross-sectional design does not allow conclusions about causality—longitudinal studies are necessary to track trajectories of disgust and contagion beliefs to better understand when and how they change throughout occupational experience. A final limitation concerns sample composition: the non-medical HW group included many members of the hygiene/infection prevention control department, who are specially trained to manage the transmission and contamination of pathogens, which may have influenced their contamination concerns and contagion beliefs. The medical HW group was also heterogeneous, comprising nurses and physicians from various disciplines exposed to different types and amounts of disgust-inducing stimuli. Further research on profession-specific differences in trait disgust and contamination beliefs could clarify whether and how occupational exposure to disgust-inducing stimuli acts as a resilience factor against C-OCD symptoms.

## 5. Conclusions

In a sample of N = 276 participants comprising psychology and medical students as well as hospital workers (HWs) with and without a medical background, disgust propensity (DP), contagion beliefs, contamination concerns, and C-OCD symptoms were assessed. In the preregistered analyses, DP was lower in medically trained groups than in psychology students and non-medical HWs, whereas contrary to our hypothesis, contagion beliefs were highest in HWs. Exploratory analyses further showed that contamination concerns were lower in medically trained participants and that, among medically trained HWs, contagion beliefs were not correlated with C-OCD symptoms. Because of the cross-sectional design, these differences cannot be attributed to medical training or hospital experience. Taken together, the findings suggest that the clinical relevance of contagion beliefs may depend less on the beliefs themselves than on their interpretation, perceived coping capacity, professional knowledge, and repeated real-world experience. This interpretation requires replication. Future research should use longitudinal designs, standardized contamination paradigms, and profession-specific analyses within the hospital context. Intervention studies could test whether strengthening perceived coping capacity and self-efficacy can refine exposure-based treatment for C-OCD.

## Figures and Tables

**Table 1:** Descriptive statistics by group. * Two psychology students identified as non-binary; thus, the number of male and female participants do not add up to the complete sample size of psychology students.

Group	Mean Age	SD Age	*n*	Female	Male
Hospital workers with medical training	43.4	12.7	111	92	19
Hospital workers without medical training	43.3	13.7	44	32	12
Medical students	24.2	3.8	69	53	16
Psychology students *	23.3	6.0	52	42	8

## Data Availability

The data presented in this study are available on request from the corresponding author due to the sensitive nature of the data.
